# Test Batteries and the Diagnostic Algorithm for Chronic Vestibular Syndromes

**DOI:** 10.3389/fneur.2021.768718

**Published:** 2021-11-18

**Authors:** Meiko Kitazawa, Yuka Morita, Chihiro Yagi, Kuniyuki Takahashi, Shinsuke Ohshima, Tatsuya Yamagishi, Shuji Izumi, Izumi Koizuka, Arata Horii

**Affiliations:** ^1^Department of Otolaryngology Head and Neck Surgery, Niigata University Graduate School of Medical and Dental Sciences, Niigata, Japan; ^2^Department of Otolaryngology, St. Marianna University School of Medicine, Kawasaki, Japan

**Keywords:** chronic vestibular syndromes, diagnosis, algorithm, persistent postural-perceptual dizziness, unilateral vestibular hypofunction, chronic dizziness due to anxiety

## Abstract

**Objective:** To develop a diagnostic algorithm for chronic vestibular syndromes by determining significant items that differ among diagnoses.

**Methods:** Two hundred thirty-one patients with chronic vestibular syndromes lasting for >3 months were included. Full vestibular tests and questionnaire surveys were performed: bithermal caloric test, cervical and ocular vestibular-evoked myogenic potential assessment, video head impulse test (vHIT), posturography, rotatory chair test, dizziness handicap inventory, hospital anxiety and depression scale (HADS), and Niigata persistent postural-perceptual dizziness (PPPD) questionnaire (NPQ). Differences in each item of the vestibular tests/questionnaires/demographic data were tested among the diagnoses. A receiver operating characteristic (ROC) curve was created for the significant items. The value that provided the best combination of sensitivity/specificity on the ROC curve was adopted as a threshold for diagnosing the targeted disease. Multiple diagnostic algorithms were proposed, and their diagnostic accuracy was calculated.

**Results:** There were 92 patients with PPPD, 44 with chronic dizziness due to anxiety (CDA), 31 with unilateral vestibular hypofunction (UVH), 37 with undifferentiated dizziness (UD), and 27 with other conditions. The top four diagnoses accounted for 88% of all chronic vestibular syndromes. Five significant items that differed among the four diseases were identified. The visual stimulation and total NPQ scores were significantly higher in the patients with PPPD than in those with UVH and UD. The percentage of canal paresis (CP %) was significantly higher in the patients with UVH than in those with PPPD, CDA, and UD. The patients with CDA were significantly younger and had higher anxiety scores on the HADS (HADS-A) than those with UVH and UD. Moreover, catch-up saccades (CUSs) in the vHIT were more frequently seen in the patients with UVH than in those with PPPD. The most useful algorithm that tested the total and visual stimulation NPQ scores for PPPD followed by the CP%/CUSs for UVH and HADS-A score/age for CDA showed an overall diagnostic accuracy of 72.8%.

**Conclusions:** Among the full vestibular tests and questionnaires, the items useful for differentiating chronic vestibular syndromes were identified. We proposed a diagnostic algorithm for chronic vestibular syndromes composed of these items, which could be useful in clinical settings.

## Introduction

The International Classification of Vestibular Diseases of the Bárány Society classifies vestibular diseases into three categories according to timing and duration of symptoms: episodic, acute, and chronic vestibular syndromes (1). In contrast to patients with acute and episodic vestibular syndromes, patients with chronic vestibular syndromes usually recover from acute symptoms and often lack remarkable findings on audio-vestibular tests. This would make diagnosing chronic vestibular syndromes difficult. Recent publications by the International Classification of Vestibular Diseases on the diagnostic criteria for chronic vestibular diseases, including persistent postural-perceptual dizziness (PPPD) (2), bilateral vestibulopathy (BVP) (3), and presbyvestibulopathy (PVP) (4), markedly accelerated our understanding of this group of diseases. Nonetheless, diagnosis of chronic vestibular syndromes, where only a few vestibular tests are useful, except for BVP and PVP, is still challenging in clinical settings. Because each chronic vestibular disease may have a different pathophysiology, these diseases should be treated with suitable strategies based on each pathophysiology, which implies that a correct diagnosis is essential.

In this study, we aimed to develop an algorithm for diagnosing chronic vestibular syndromes. For this purpose, we conducted full vestibular tests, including bithermal caloric test, cervical, and ocular vestibular-evoked myogenic potential (cVEMP and oVEMP, respectively) assessment, video head impulse test (vHIT), posturography, and rotatory chair test, and several questionnaire surveys for vestibular symptoms and mental disorders, such as the Dizziness Handicap Inventory (DHI), Hospital Anxiety and Depression Scale (HADS), and Niigata PPPD Questionnaire (NPQ), for patients with chronic vestibular symptoms lasting for >3 months. Differences in the results of each test were compared among chronic vestibular syndromes. Several significant test items were then extracted as useful test batteries for the diagnosis of chronic vestibular syndromes. Finally, several diagnostic algorithms using combinations of the test batteries were proposed, and the diagnostic accuracy of each algorithm was compared among the algorithms.

## Materials and Methods

This study was approved by the Institutional Review Board of Niigata University Hospital (No. 2020-0445) and was conducted in accordance with the Declaration of Helsinki.

### Patients

We retrospectively examined the medical records of patients with chronic vestibular symptoms lasting for >3 months between October 2016 and September 2019. There were 231 participants: 150 women and 81 men with a median age of 52 years (range: 10–90 years).

### Diagnosis

PPPD, BVP, and PVP were all diagnosed in accordance with the Bárány Society criteria (2–4). Unilateral vestibular hypofunction (UVH) was defined as unilateral abnormal values in the caloric test and/or vHIT, with a clear history of acute vertigo spell (5). Chronic dizziness due to anxiety (CDA) was defined as chronic dizziness with typical psychiatric and somatic symptoms of anxiety. If the cause of vestibular symptoms could not be specified, the patient was classified as having undifferentiated dizziness (UD).

### Caloric Test

An alternate bithermal (26 and 45°C) air caloric test was performed. The maximum slow-phase velocity of the nystagmus was measured using electronystagmography, and the percentage of canal paresis (CP%) was calculated using the formula of Jongkees et al. (6). A CP% of >20% was considered to indicate significant unilateral caloric weakness. The percentage of directional preponderance was also calculated.

### VEMP

To quantify otolithic function, we recorded the cVEMP and oVEMP using the Neuropack System (Nihon Kohden, Japan). Click (0.1-ms rarefactive square waves of 105-dB nHL) was used to induce the cVEMP. Meanwhile, a hand-held electromechanical vibrator (Minishaker, Bruel & Kjaer, Denmark) fitted with a short bolt terminating in a plastic cap was used to record the oVEMP. The vibrator delivered a 500-Hz tone burst (4-ms plateau and 1-ms rise and fall) on the subjects' skull at the Fz (midline of the hairline). The interaural asymmetry ratios (IAARs) of the cVEMP and oVEMP were obtained using the following formula:

IAAR = (Ar – Al)/(Ar + Al) × 100

Ar: normalized amplitude (p13-n23 or n10-p15) on the right side.

Al: normalized amplitude (p13-n23 or n10-p15) on the left side.

A |AR| of >33.3% was defined as unilateral saccular (cVEMP) or utricular (oVEMP) dysfunction.

### Rotatory Chair Test

The rotatory chair test was performed using Nistamo21 IRN 2 (Morita, Japan). Therein, the patients sat in a rotatory chair to which a pendulum-like rotation was applied, so that the maximum head angular velocity was 50°/s at a stimulation frequency of 0.1 Hz. The angular velocity of eye movements was monitored and analyzed. The vestibulo-ocular reflex directional preponderance was calculated, and a value of >12% was considered significant.

### Posturography

The patients underwent static posturography on a solid or rubber foam surface using Gravicoda^®^ (ANIMA Corp., Japan), with their eyes opened and closed. The recording time was 60 s or until the subjects required assistance to prevent falling. In the eyes-open condition, the subjects were asked to watch a small, red circle 2 m away from where they were standing in a quiet, well-lit room. In the eyes-closed condition, the foam ratio (posturography with/without foam) was used as an indicator of somatosensory dependence of postural control and the Romberg ratio on the foam as an indicator of visual dependence.

### DHI

The DHI is a standard questionnaire that quantitatively evaluates the degree of handicap in the daily life of patients with vestibular disorders and consists of 25 questions (7, 8). The total score ranges from 0 (no disability) to 100 (severe disability).

### HADS

The HADS is a 14-item questionnaire comprising two subscales for assessing non-somatic symptoms of anxiety and depression. Each item in the questionnaire is rated from 0 to 3. The scores on the two subscales range from 0 (no sign of anxiety or depression) to 21 (maximum level of anxiety or depression). A score of ≥11 indicates clinically significant anxiety or depressive symptoms (9).

### NPQ

The NPQ evaluates the degree of symptom exacerbation of PPPD using three characteristic factors: upright posture/walking, movement, and visual stimulation (10). Each question is scored from 0 (none) to 6 (unbearable); therefore, the total possible score for each factor is 24, and the total possible score for all three factors is 72.

### Test Batteries and Diagnostic Algorithms

As shown later in the results section, four top diagnoses (PPPD, CDA, UVH, and UD) accounted for 88% of all chronic vestibular syndromes. Therefore, we concentrated on these four diseases in the later process of building diagnostic algorithms. Differences in the demographic data and results of the vestibular tests and questionnaires were compared among the four diseases using analysis of variance (Kruskal–Wallis test). The *post-hoc* Dann Bonferroni test was followed for the positive items.

As test batteries for diagnosing chronic vestibular syndromes, statistically significant results of vestibular tests, validated total and subscale scores of questionnaires, and demographic data were extracted. Receiver operating characteristic (ROC) curves for each item were created to diagnose the targeted disease. The accuracy for predicting the targeted disease was estimated using the area under the curve (AUC) of the ROC curve. Based on the ROC curves, threshold values to obtain the best combination of sensitivity and specificity were calculated. These thresholds were adopted when diagnosing the targeted disease using the following diagnostic algorithms. Multiple algorithms composed of the same positive items but with different orders were proposed for diagnosing chronic vestibular syndromes. Finally, the diagnostic accuracies of the algorithms were compared. Owing to the retrospective nature of the study, there was a lack of data for the vestibular tests, and only 118 out of the 231 patients who received all test batteries were enrolled in the analysis of the algorithms.

Given that the CP% was significantly higher in the patients with UVH than in those with PPPD (see results section), the vHIT was additionally performed for a limited number of these patients. The vHIT was performed using EyeSeeCam (Interacoustics, 5500 Middelfart, Denmark). The vestibulo-ocular reflex was generated through the rotation of the subjects' head, unpredictable in direction and time (peak head velocity of 150/s to 300/s), by the examiner who stood behind the subjects. The head impulses were delivered in the horizontal plane. Each participant underwent a minimum of five head impulses. Vestibulo-ocular reflex gains for the right and left lateral canals (RL and LL, respectively) were measured at 60 ms. The following vHIT parameters were analyzed and compared among the diseases: vestibulo-ocular reflex gain on the better and worse sides, asymmetry ratio (AR), and presence of catch-up saccades (CUSs, overt, and/or covert). The AR was calculated using the following formula: AR = |(RL–LL)/(RL+LL)| ×100 (%).

### Statistics

Statistical analyses were conducted using the SPSS version 26 software. Statistical significance was set at a threshold of *p* < 0.05.

## Results

The specific diagnoses of the 231 patients were as follows: PPPD (*n* = 92), CDA (*n* = 44), UVH (*n* = 31), UD (*n* = 37), and other conditions (*n* = 27). The last 27 patients included 7 with BVP, 8 with brain tumor, 4 with PVP, 2 with spinocerebellar ataxia, 2 with fracture of the temporal bone, 2 with orthostatic dysfunction,1 with superior canal dehiscence syndrome, and 1 with petrous apex cholesterol granuloma. Since the top four diagnoses (PPPD, CDA, UVH, and UD) accounted for 88% of the chronic vestibular diseases (*n* = 204/231), we concentrated on these four diagnoses when deciding the test batteries for diagnosing chronic vestibular syndromes.

**Figure 1:**
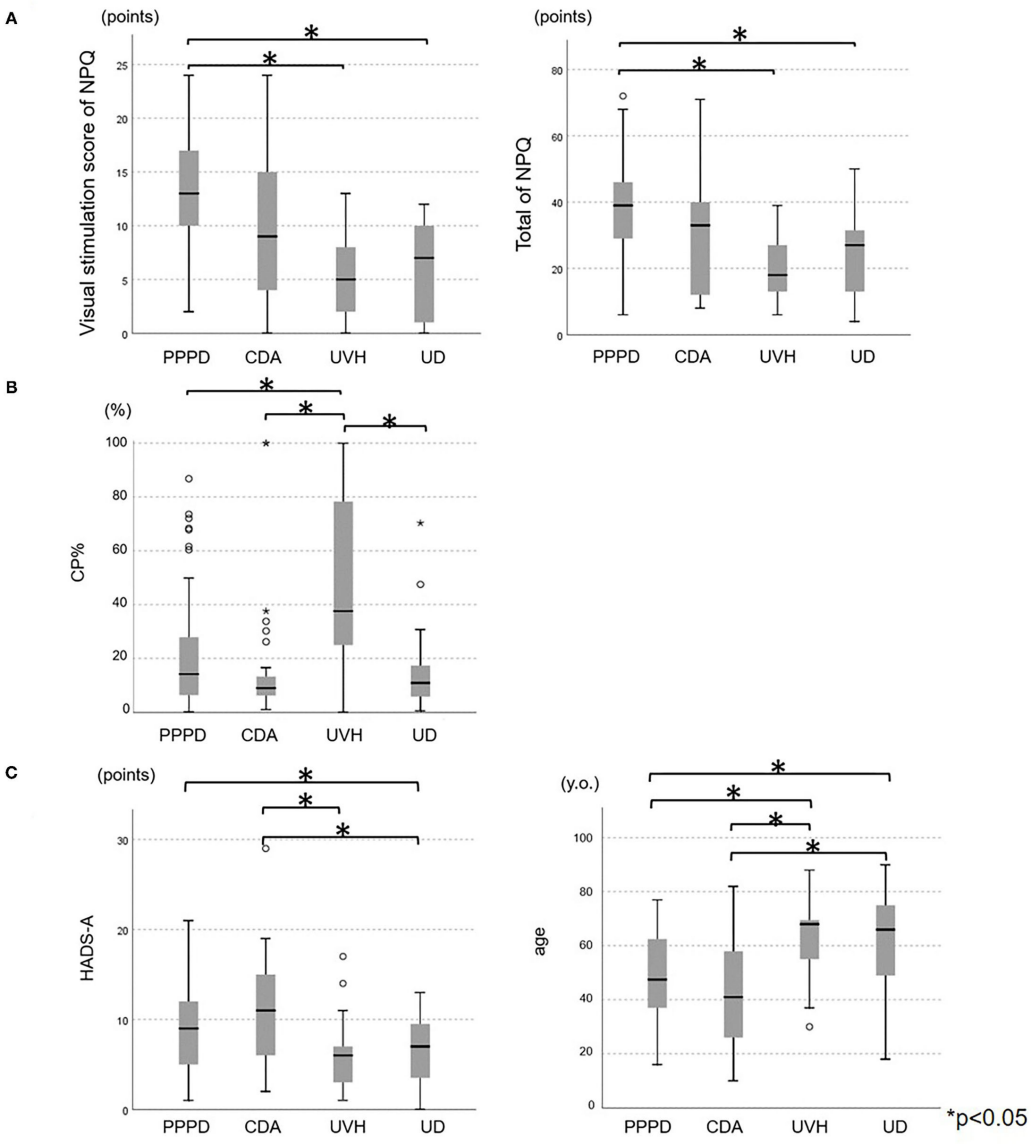
Comparisons of the **(A)** NPQ scores, **(B)** CP%, and **(C)** HADS-A score and age among the chronic vestibular syndromes. **(A)** The visual stimulation score of the NPQ of the PPPD group (13.05 ± 5.21) was significantly higher than that of the UVH (5.27 ± 4.08) (*p*<0.05) and UD groups (6.20 ± 4.78) (*p*<0.05). The total score of the NP of the PPPD group (37.70 ± 13.51) was significantly higher than that of the UVH (19.80 ± 9.95) (*p*<0.05) and UD groups (24.20 ± 14.90) (*p*<0.05). **(B)** The CP% of the UVH group (51.81 ± 32.38) was significantly higher than that of the PPPD group (20.17 ± 19.59) (*p*<0.05), CDA group (16.57 ± 22.82) (*p*<0.05), and UD group (14.94 ± 15.25) (*p*<0.05). **(C)** The HADS-A score of the CDA group (11.15 ± 5.89) was significantly higher than that of the UVH group (6.04 ± 3.85) (*p*<0.05) and UD group (6.19 ± 3.53) (*p*<0.05). The HADS-A score of the PPPD group (9.18 ± 4.70) was significantly higher than that of the UD group (6.19 ± 3.53) (*p*<0.05). The patients in the CDA group (42.4 ± 18.9 years) were significantly younger than those in the UVH group (63.5 ± 13.3 years) (*p*<0.05) and UD group (60.2 ± 19.7 years) (*p*<0.05). The patients in the PPPD group (48.3 ± 15.2 years) were significantly younger than those in the UVH group (63.5 ± 13.3 years) (*p*<0.05) and UD group (60.2 ± 19.7 years) (*p*<0.05). NPQ, Niigata PPPD questionnaire; CP%, percentage of canal paresis; HADS-A, anxiety score on the hospital anxiety and depression scale; PPPD, persistent postural-perceptual dizziness; CDA, chronic dizziness due to anxiety; UVH, unilateral vestibular hypofunction; UD, undifferentiated dizziness.

Among the five vestibular tests, three questionnaires, and demographic data, 12 items were significantly different among the four disease groups based on the Kruskal–Wallis test findings (Table 1). The *post-hoc* Dann Bonferroni test revealed 5 significant items out of the 12 items: visual stimulation score and total score of the NPQ, CP%, anxiety score on the HADS (HADS-A), and age (Figure 1). The visual stimulation score of the NPQ of the patients with PPPD (13.05 ± 5.21) was significantly higher than that of those with UVH (5.27 ± 4.08) (*p* < 0.05) and UD (6.20 ± 4.78) (*p* < 0.05; Figure 1A). The total score of the NPQ of the patients with PPPD (37.70 ± 13.51) was also significantly higher than that of those with UVH (19.80 ± 9.95) (*p* < 0.05) and UD (24.20 ± 14.90) (*p* < 0.05; Figure 1A). The CP% of the patients with UVH (51.81 ± 32.38%) was significantly higher than that of those with PPPD (20.17 ± 19.59%) (*p* < 0.05), CDA (16.57 ± 22.82%) (*p* < 0.05), and UD (14.94 ± 15.25%) (*p* < 0.05; Figure 1B). The HADS-A score of the patients with CDA (11.15 ± 5.89) was significantly higher than that of those with UVH (6.04 ± 3.85) (*p* < 0.05) and UD (6.19 ± 3.53) (*p* < 0.05; Figure 1C). The HADS-A score of the patients with PPPD (9.18 ± 4.70) was significantly higher than that of those with UD (6.19 ± 3.53) (*p* < 0.05; Figure 1C). The patients with CDA (42.4 ± 18.9 years) were significantly younger than those with UVH (63.5 ± 13.3 years) (*p* < 0.05) and UD (60.2 ± 19.7 years) (*p* < 0.05; Figure 1C). The patients with PPPD (48.3 ± 15.2 years) were significantly younger than those with UVH (63.5 ± 13.3 years) (*p* < 0.05) and UD (60.2 ± 19.7 years) (*p* < 0.05; Figure 1C). No items could discriminate between the patients with PPPD and CDA.

**Table 1:** Differences in demographic data and results of vestibular tests and questionnaires compared between the four diseases by analysis of variance (Kruskal-Wallis test).

**Variables**		**PPPD**	**CDA**	**UVH**	**UD**	***p*** **-value**
		**Mean ± SD**	**Mean ± SD**	**Mean ± SD**	**Mean ± SD**	
**Age (y.o.)**		48.3 ± 15.2	42.4 ± 18.9	63.5 ± 13.3	60.2 ± 19.7	**<0.001**
**HADS**	**Anxiety**	9.18 ± 4.70 (*n* = 87)	11.15 ± 5.89 (*n* = 34)	6.04 ± 3.85 (*n* = 25)	6.19 ± 3.53 (*n* = 28)	**<0.001**
	Depression	8.31 ± 4.18 (*n* = 87)	8.88 ± 3.12 (*n* = 34)	7.76 ± 3.93 (*n* = 25)	6.75 ± 3.64 (*n* = 28)	0.073
	**Total**	17.49 ± 7.98 (*n* = 87)	19.2 ± 6.61 (*n* = 34)	13.80 ± 7.26 (*n* = 25)	13.04 ± 5.99 (*n* = 28)	**0.001**
**DHI**	**Physical**	15.75 ± 6.00 (*n* = 87)	12.29 ± 6.79 (*n* = 34)	12.08 ± 5.97 (*n* = 26)	10.00 ± 5.42 (*n* = 28)	**0.001**
	Emotional	19.36 ± 8.22 (*n* = 87)	17.29 ± 8.75 (*n* = 34)	14.85 ± 6.48 (*n* = 26)	14.43 ± 8.19 (*n* = 28)	0.091
	**Functional**	19.26 ± 9.50 (*n* = 87)	17.94 ± 9.31 (*n* = 34)	14.08 ± 8.49 (*n* = 26)	11.64 ± 8.96 (*n* = 28)	**0.029**
	**Total**	54.37 ± 20.80 (*n* = 87)	44.82 ± 21.56 (*n* = 34)	41.00 ± 17.29 (*n* = 26)	36.07 ± 19.76 (*n* = 28)	**0.007**
**NPQ**	**Upright posture/walking**	11.86 ± 5.78 (*n* = 77)	10.05 ± 6.89 (*n* = 20)	7.13 ± 5.11 (*n* = 15)	9.07 ± 5.69 (*n* = 15)	**0.02**
	**Movement**	12.94 ± 4.84 (*n* = 77)	10.95 ± 6.06 (*n* = 20)	7.40 ± 4.12 (*n* = 15)	8.93 ± 5.43 (*n* = 15)	**0.002**
	**Visual stimulation**	13.05 ± 5.21 (*n* = 77)	9.90 ± 6.94 (*n* = 20)	5.27 ± 4.08 (*n* = 15)	6.20 ± 4.78 (*n* = 15)	**<0.001**
	**Total**	37.70 ± 13.51 (*n* = 77)	30.90 ± 18.00 (*n* = 20)	19.80 ± 9.95 (*n* = 15)	24.20 ± 14.90 (*n* = 15)	**<0.001**
**Caloric test**	**CP (%)**	20.17 ± 19.59 (*n* = 83)	16.57 ± 22.82 (*n* = 34)	51.81 ± 32.38 (*n* = 23)	14.94 ± 15.25 (*n* = 30)	**<0.001**
	**DP (%)**	15.20 ± 14.55 (*n* = 83)	10.17 ± 11.98 (*n* = 34)	17.98 ± 14.54 (*n* = 23)	15.53 ± 11.53 (*n* = 30)	**0.004**
cVEMP	Asymmetry ratio (%)	5.52 ± 33.87 (*n* = 89)	10.56 ± 34.44 (*n* = 43)	20.69 ± 38.70 (*n* = 24)	1.62 ± 46.80 (*n* = 28)	0.464
oVEMP	Asymmetry ratio (%)	−1.30 ± 31.92 (*n* = 91)	−4.13 ± 25.38 (*n* = 43)	−1.27 ± 44.20 (*n* = 24)	−5.12 ± 38.37 (*n* = 28)	0.596
Posturography	Foam ratio	2.05 ± 0.61 (*n* = 85)	1.95 ± 0.53 (*n* = 35)	2.40 ± 1.29 (*n* = 23)	2.18 ± 0.64 (*n* = 30)	0.264
	Romberg ratio on foam	1.85 ± 0.59 (*n* = 85)	1.82 ± 0.55 (*n* = 35)	2.00 ± 0.64 (*n* = 23)	2.51 ± 3.40 (*n* = 30)	0.226
Rotatory chair test	VOR-DP (%)	3.85 ± 17.79 (*n* = 87)	−1.69 ± 15.85 (*n* = 41)	1.66 ± 25.75 (*n* = 28)	−0.50 ± 14.07 (*n* = 36)	0.394

*The bold values represents the statistically significant differences*.

**Figure 2:**
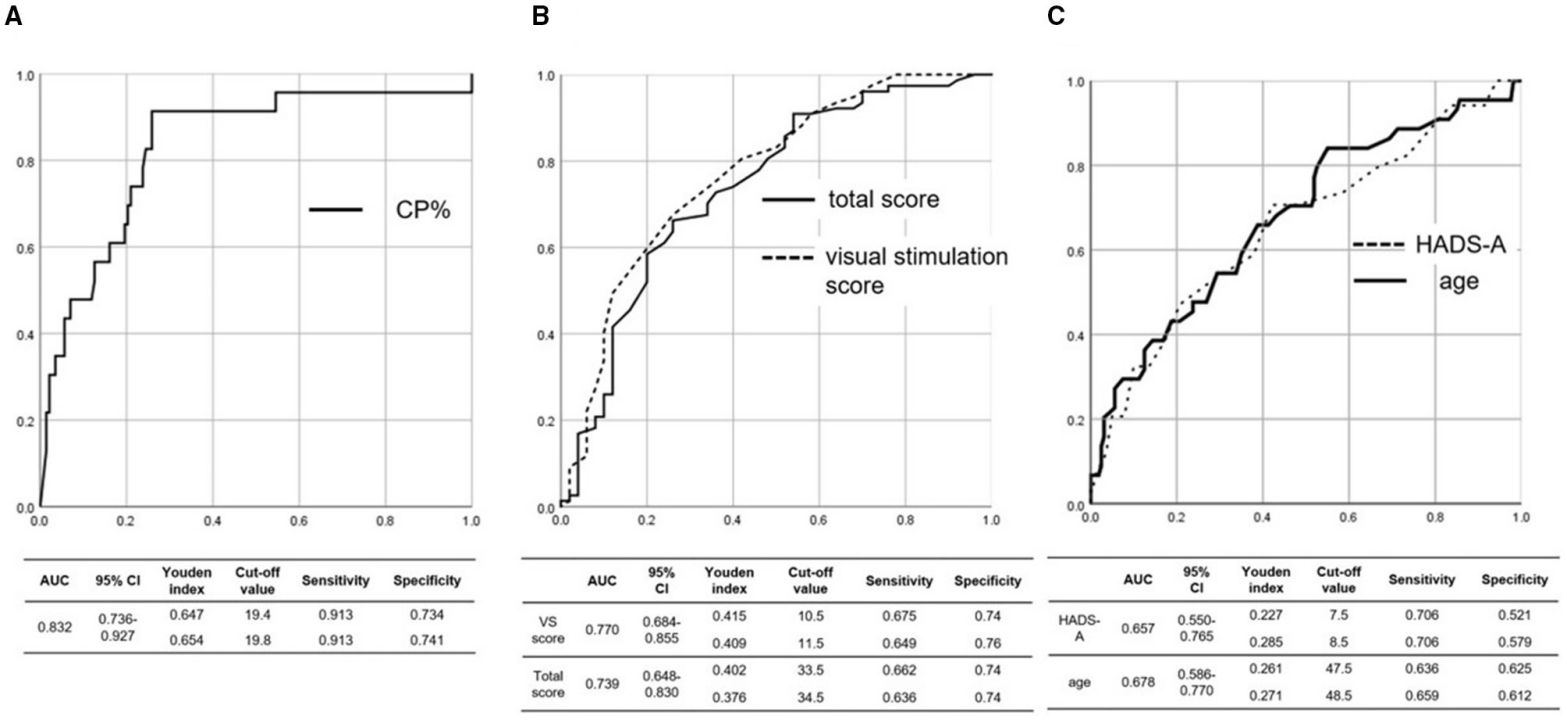
ROC curve for the **(A)** CP%, **(B)** total and visual stimulation scores of the NPQ, and **(C)** age and HADS-A score. **(A)** The AUC of the ROC curve for the CP% was 0.832, and a CP% of 19.8 had the best sensitivity (91.3%) and specificity (74.1%) for diagnosing UVH. **(B)** The AUC of the ROC curve for the visual stimulation score and total score of the NPQ was 0.770 and 0.739, respectively, and visual stimulation score and total score of the NPQ of 10.55 and 33.5, respectively, had the best sensitivity (visual stimulation score of 10.5: 67.5%; total score of 33.5: 66.2%) and specificity (visual stimulation score of 10.5: 74.0%; total score of 33.5: 74.0%) for diagnosing PPPD. **(C)** The AUC of the ROC curve for age and the HADS-A score was 0.678 and 0.657, respectively, and age and HADS-A score of 48.5 years and 8.5, respectively, had the best sensitivity (age of 48.5 years: 65.9%; HADS-A score of 8.5: 70.6%) and specificity (age of 48.5 years: 61.2%; HADS-A score of 8.5: 57.9%) for diagnosing CDA. ROC, receiver operating characteristic; NPQ, Niigata PPPD questionnaire; CP%, percentage of canal paresis; HADS-A, anxiety score on the hospital anxiety and depression scale; AUC, area under the curve; PPPD, persistent postural-perceptual dizziness; CDA, chronic dizziness due to anxiety; UVH, unilateral vestibular hypofunction; CI, confidence interval.

The AUC, 95% confidence interval (CI), and Youden index, which implies the best combination of sensitivity and specificity of ROC curves, are shown in Figure 2. The AUC of the ROC curve for the CP% was 0.832 (95% CI: 0.736–0.927), and a CP% of 19.8 had the best sensitivity (91.3%) and specificity (74.1%) for diagnosing UVH (Figure 2A). The AUC of the ROC curve for the visual stimulation score and total score of the NPQ was 0.770 (95% CI: 0.684–0.855) and 0.739 (95% CI: 0.648–0.830), respectively, and visual stimulation score and total score of the NPQ of 10.5 and 33.5, respectively, had the best sensitivity (visual stimulation score of 10.5: 67.5%; total score of 33.5: 66.2%) and specificity (visual stimulation score of 10.5: 74.0%; total score of 33.5: 74.0%) for diagnosing PPPD (Figure 2B). The AUC of the ROC curve for the HADS-A score and age was 0.657 (95% CI: 0.550–0.765) and 0.678 (95% CI: 0.586–0.770), respectively, and HADS-A score and age of 8.5 and 48.5 years, respectively, had the best sensitivity (HADS-A score of 8.5: 70.6%; age of 48.5 years: 65.9%) and specificity (HADS-A score of 8.5: 57.9%; age of 48.5 years: 61.2%) for diagnosing CDA (Figure 2C).

Given that the CP% was useful for diagnosing UVH (AUC: 0.832), the vHIT, which is more accessible and widely used, was additionally performed for a limited number of patients with UVH and PPPD. As shown in Table 2, CUSs were more frequently seen in the patients with UVH than in those with PPPD (*p* = 0.002), while the other variables, including vHIT gain on both better and worse sides, AR, and number of patients showing vHIT gains of < 0.6, were not different between the patients with UVH and PPPD.

**Table 2:** V-HIT results for PPPD and UVH.

**Variables**	**PPPD (*n* = 56)**	**UVH (*n* = 6)**	***p*** **-value**
	**Mean ± SD**	**Mean ± SD**	
V-HIT (better)	0.98 ± 1.53	0.89 ± 0.36	0.898
V-HIT (worse)	0.82 ± 0.19	0.67 ± 0.31	0.276
V-HIT (asymmetry ratio, %)	9.83 ± 10.27	14.44 ± 11.09	0.276
V-HIT gain < 0.6	7 (12.5%)	2 (33.3%)	0.206
Catch up saccade (+)	1 (1.8%)	3 (50%)	0.002

Based on the significance of the abovementioned results, the visual stimulation score and total score of the NPQ were adopted as the items that could discriminate PPPD from the other chronic vestibular syndromes. The CP% and presence of CUSs in the vHIT were used to diagnose UVH. The HADS-A score and age were adopted as the criteria for diagnosing CDA. While the HADS-A score and age in the patients with PPPD significantly differed from those in the patients with UVH and UD (Figure 1), the AUC for the NPQ scores was higher than that for the HADS-A score and age (Figure 2). Therefore, the NPQ scores (total score and visual stimulation score) rather than the HADS-A score or age were used to diagnose PPPD.

Diagnosing PPPD, the most frequent disease, was prioritized in Algorithms 1 and 2 using the NPQ items first. In contrast, assessment of the presence of CUSs in the vHIT and CP%, the most accurate item (highest AUC of the ROC curves), which can discriminate UVH from the other diseases, was prioritized in Algorithms 3 and 4. Diagnosing CDA first was prioritized in Algorithms 5 and 6.

**Figure 3:**
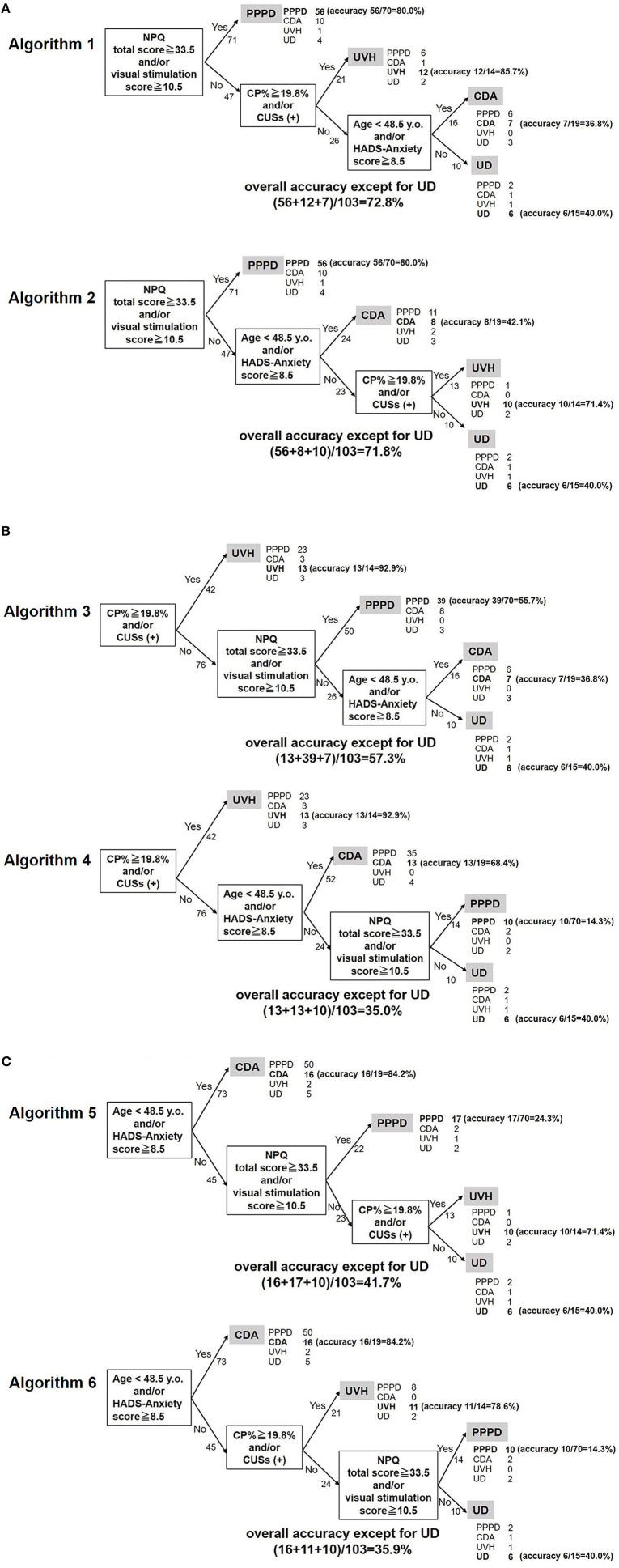
Diagnostic algorithms for chronic vestibular syndromes. **(A)** Algorithms diagnosing PPPD first. The overall accuracies for Algorithms 1 and 2 were 72.8 and 71.8%, respectively. **(B)** Algorithms diagnosing UVH first. The overall accuracies for Algorithms 3 and 4 were 57.3 and 35.0%, respectively. **(C)** Algorithms diagnosing CDA first. The overall accuracies for Algorithms 5 and 6 were 41.7 and 35.9%, respectively. NPQ, Niigata PPPD questionnaire; CP%, percentage of canal paresis; HADS, hospital anxiety and depression scale; PPPD, persistent postural-perceptual dizziness; CDA, chronic dizziness due to anxiety; UVH, unilateral vestibular hypofunction; UD, undifferentiated dizziness; CUS, catch-up saccade.

Figure 3A (upper panel) shows the diagnostic accuracy for each disease group using Algorithm 1: 80, 85.7, and 36.8% of the PPPD, UVH, and CDA cases, respectively, were correctly diagnosed. Figure 3A (lower panel) shows the diagnostic accuracy for each disease group using Algorithm 2: 80, 71.4, and 42.1% of the PPPD, UVH, and CDA cases, respectively, were correctly diagnosed. Figure 3B (upper panel) shows the diagnostic accuracy for each group using Algorithm 3: 92.9, 55.7, and 36.8% of the UVH, PPPD, and CDA cases, respectively, were correctly diagnosed. Figure 3B (lower panel) shows the diagnostic accuracy for each group using Algorithm 4: 92.9, 14.3, and 68.4% of the UVH, PPPD, and CDA cases, respectively, were correctly diagnosed. Figure 3C (upper panel) shows the diagnostic accuracy for each group using Algorithm 5: 84.2, 24.3, and 71.4% of the CDA, PPPD, and UVH cases, respectively, were correctly diagnosed. Figure 3C (lower panel) shows the diagnostic accuracy for each group using Algorithm 6: 84.2, 14.3, and 78.6% of the CDA, PPPD, and UVH cases, respectively, were correctly diagnosed. The final diagnostic accuracies for all diseases, except for UD, were 72.8% for Algorithm 1, 71.8% for Algorithm 2, 57.3% for Algorithm 3, 35.0% for Algorithm 4, 41.7% for Algorithm 5, and 35.9% for Algorithm 6.

## Discussion

The International Classification of Vestibular Diseases of the Bárány Society classifies vestibular diseases into episodic, acute, and chronic vestibular syndromes. PPPD, BVP, UVH, PVP, psychogenic dizziness, chronic stage of stroke, and other peripheral and central diseases can cause chronic vestibular syndromes. Patients with chronic vestibular syndromes usually recover from acute symptoms and often lack distinguishable symptoms or findings on vestibular tests. Nonetheless, each chronic vestibular disease may have a different pathophysiology and should therefore be treated using different strategies (11–13). As the first step in the correct diagnosis of chronic vestibular diseases, information on disease distribution is important. As mentioned in the Results section, PPPD was the most frequent disease, followed by CDA, UVH, and UD. These four diagnoses accounted for 88% of the chronic vestibular syndromes. Although other chronic diseases, such as BVP and PVP, are of importance in clinical settings, it would be reasonable to target the four major diagnoses when building test batteries and diagnostic algorithms for screening chronic vestibular syndromes. Based on these disease distributions, six algorithms for diagnosing PPPD, UVH, and CDA were proposed (Figure 3).

The Kruskal–Wallis test for several items, such as age, HADS-A score, total HADS score, physical DHI score, functional DHI score, total DHI score, upright posture/walking NPQ score, movement NPQ score, visual stimulation NPQ score, total NPQ score, CP%, and percentage of directional preponderance, revealed significant differences among the disease groups (Table 1); however, the *post-hoc* Dann Bonferroni test demonstrated that only five factors were significantly different among them: visual stimulation and total scores of the NPQ, CP%, HADS-A score, and age (Figure 1). In addition, the presence of CUSs in the vHIT was useful for discriminating UVH from PPPD (Table 2). Based on these results, the visual stimulation and total scores of the NPQ, CP%/CUSs, HADS-A score, and age were selected as the test batteries for screening for the chronic vestibular syndromes. For screening purposes, the availability of each test in clinical settings is important: Four of the six selected items can be obtained using questionnaires and demographic data assessment. The remaining two items were the CP% and presence of CUSs in the vHIT. Although bithermal caloric tests are not necessarily available at all clinics, the vHIT (presence of CUSs), which is becoming an alternative for caloric tests, is currently widely performed in most outpatient clinics. Therefore, diagnostic algorithms created on the basis of these items would be suitable for screening purposes in clinical settings.

Central disorders, such as stroke, should be prioritized in emergent clinical settings when diagnosing acute vestibular syndromes. For this purpose, bedside inspection, namely HINTS, has been widely accepted as a screening tool in emergency outpatient departments (14). In contrast, there are no widely accepted algorithms for the screening of chronic vestibular syndromes. Recent publications on the diagnostic criteria for chronic vestibular syndromes, including PPPD, BVP, and PVP, have greatly improved the understanding and treatment of this group of syndromes. Nonetheless, a large number of vestibular tests and questionnaire surveys are routinely performed for patients with chronic vestibular disorders, most of which reveal no apparent abnormalities. From the viewpoint of cost effectiveness, it is ideal to extract useful screening items for diagnosing chronic vestibular syndromes and to establish a useful diagnostic algorithm.

Six algorithms for diagnosing chronic vestibular syndromes based on the abovementioned significant items with different orders were proposed (Figures 3A–C). Diagnosing PPPD, the most frequent disease, was prioritized in Algorithms 1 and 2 using the NPQ items first. In contrast, assessment of the CP% and presence of CUSs, which can discriminate UVH from the other diseases, was prioritized in Algorithms 3 and 4. Diagnosing CDA first was prioritized in Algorithms 5 and 6.

Because the final diagnostic accuracy for all diseases in Algorithms 1 and 2 (72.8 and 71.8%, respectively) was better than that in Algorithms 3 and 4 (57.3 and 35.0%, respectively) and Algorithms 5 and 6 (41.7 and 35.9%, respectively), Algorithms 1 and 2, which prioritize the diagnosis of the most frequent chronic vestibular syndrome (PPPD), may be the best tools for screening purposes. According to the diagnostic criteria of PPPD, it may co-exist with other diseases or disorders, and evidence of another active illness does not necessarily exclude a diagnosis of PPPD (2). Therefore, Algorithms 3–6, which diagnose possible comorbid diseases (UVH or CDA) first, may have the risk of missing PPPD. This point also suggests that Algorithms 1 and 2, which diagnose PPPD first, may be the most suitable screening algorithms for chronic vestibular syndromes. Given that the diagnostic algorithm could be proposed, it could be possibly expected to the machine learning/artificial intelligence in the future. However, overall accuracy of the Algorithm 1 was up to 72.8%, which is not still sufficient. Developing a tool for discriminating between PPPD and CDA may improve the diagnostic accuracy of the algorithms.

Although the accuracy for diagnosing PPPD and UVH was within the satisfactory levels (71.4–85.7%) in both Algorithms 1 and 2, the accuracy for diagnosing CDA was as low as 36.8–42.1% (Figure 3A). This is because 10 of the 19 patients with CDA were misdiagnosed with PPPD in Algorithms 1 and 2 (Figure 3A). While Algorithms 1 and 2 are recommended as screening tools for patients with chronic vestibular disorders, those with significant NPQ items in these algorithms should be carefully interviewed regarding the characteristics of their symptoms and exacerbating factors of PPPD to correctly diagnose this disease.

Patients with chronic vestibular syndromes sometimes have comorbid chronic and/or episodic syndromes which might potentially affect the performance of diagnostic algorithms. Regarding the comorbidity between chronic vestibular syndromes, there were four patients with PPPD/UVH and also four patients with PPPD/CDA. Similarly, there were six patients with PPPD/Meniere's disease, one patient with CDA/Meniere's disease, and one patient with UD/BPPV (data not shown). According to the diagnostic criteria (2), we diagnosed PPPD only when the core vestibular symptoms and exacerbating factors seen in the patient could not be explained by the comorbid disease such as UVH, CDA, and Meniere's disease. In other words, we did not diagnose PPPD if the core vestibular symptoms and exacerbating factors could be accounted for by the comorbid disease. Therefore, the comorbidity between PPPD and chronic/episodic syndromes did not affect the performance of the algorithms as long as diagnosing PPPD strictly based on the diagnostic criteria (2). Regarding the comorbidity between CDA/UD and episodic syndromes, we diagnosed CDA/UD based on the patients' chronic symptoms and any comorbid episodic symptoms did not affect the diagnosis of CDA/UD. Therefore, the comorbidity between the chronic and episodic syndromes did not affect the performance of the algorithm.

### Limitations

PPPD, UVH, and CDA are not the only diseases that cause chronic vestibular syndromes. The proposed algorithm may be helpful but is not an absolute tool for diagnosing chronic vestibular syndromes. This study had a retrospective design, and prospective multicenter trials are necessary for validating the accuracy of the algorithm.

### Conclusions

The items that are useful for differentiating between chronic vestibular diseases were identified: visual stimulation and total scores of the NPQ, CP% and presence of CUSs in the vHIT, HADS-A score, and age. We proposed a diagnostic algorithm for chronic vestibular diseases using these items, which showed an overall accuracy of 72.8%. Although the final diagnosis must be based on the diagnostic criteria, this algorithm may be helpful for screening purposes.

## Data Availability Statement

The raw data supporting the conclusions of this article will be made available by the authors, without undue reservation.

## Ethics Statement

The studies involving human participants were reviewed and approved by Niigata University Hospital (No. 2020-0445). Written informed consent from the participants' legal guardian/next of kin was not required to participate in this study in accordance with the national legislation and the institutional requirements.

## Author Contributions

MK and YM conceived the study, collected and analyzed the data, and wrote the manuscript. AH and IK directed the project. CY, KT, SO, TY, and SI collected the data. All authors participated in the data discussion. All authors contributed to the article and approved the submitted version.

## Funding

This work was supported by Grants-in-Aid for Scientific Research (B) (PI: AH) and (C) (PI: CY and SO) from the Ministry of Education, Culture, Sports, Science and Technology of Japan, and Japan Agency for Medical Research and Development (AMED, PI: IK).

## Conflict of Interest

The authors declare that the research was conducted in the absence of any commercial or financial relationships that could be construed as a potential conflict of interest.

## Publisher's Note

All claims expressed in this article are solely those of the authors and do not necessarily represent those of their affiliated organizations, or those of the publisher, the editors and the reviewers. Any product that may be evaluated in this article, or claim that may be made by its manufacturer, is not guaranteed or endorsed by the publisher.
